# 2,2′-[2,4-Bis(naphthalen-1-yl)cyclo­butane-1,3-di­yl]bis­(1-methyl­pyridinium) diiodide: thermal-induced [2 + 2] cyclo­addition reaction of a heterostilbene^1^

**DOI:** 10.1107/S1600536811052433

**Published:** 2011-12-10

**Authors:** Suchada Chantrapromma, Kullapa Chanawanno, Nawong Boonnak, Hoong-Kun Fun

**Affiliations:** a Crystal Materials Research Unit, Department of Chemistry, Faculty of Science, Prince of Songkla University, Hat-Yai, Songkhla 90112, Thailand; b X-ray Crystallography Unit, School of Physics, Universiti Sains Malaysia, 11800 USM, Penang, Malaysia

## Abstract

The asymmetric unit of the title compound, C_36_H_32_N_2_^2+^·2I^−^, consists of one half-mol­ecule of the cation and one I^−^ anion. The cation is located on an inversion centre. The dihedral angle between the pyridinium ring and the naphthalene ring system in the asymmetric unit is 19.01 (14)°. In the crystal, the cations and the anions are linked by C—H⋯I inter­actions into a layer parallel to the *bc* plane. Intra- and inter­molecular π–π inter­actions with centroid–centroid distances of 3.533 (2)–3.807 (2) Å are also observed.

## Related literature

For bond-length data, see: Allen *et al.* (1987 ▶). For background to stilbene and [2 + 2] photodimerization, see: Chanawanno *et al.* (2010 ▶); Papaefstathiou *et al.* (2002 ▶); Ruanwas *et al.* (2010 ▶); Yayli *et al.* (2004 ▶). For related structures, see: Fun, Chanawanno & Chantrapromma (2009 ▶); Fun, Surasit *et al.* (2009 ▶). For the stability of the temperature controller used in the data collection, see: Cosier & Glazer (1986 ▶).
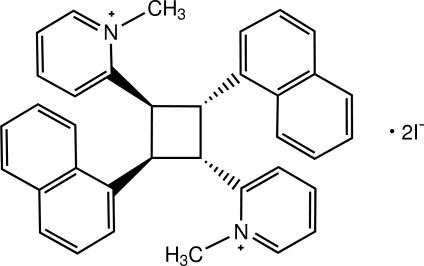


## Experimental

### 

#### Crystal data

C_36_H_32_N_2_^2+^·2I^−^
*M* *_r_* = 746.44
Monoclinic, 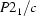

*a* = 7.0061 (1) Å
*b* = 20.7920 (4) Å
*c* = 10.8956 (2) Å
β = 106.063 (1)°
*V* = 1525.21 (5) Å^3^
*Z* = 2
Mo *K*α radiation
μ = 2.09 mm^−1^
*T* = 100 K
0.15 × 0.13 × 0.08 mm

#### Data collection

Bruker APEXII CCD area-detector diffractometer
Absorption correction: multi-scan (*SADABS*; Bruker, 2005 ▶) *T*_min_ = 0.749, *T*_max_ = 0.854
18762 measured reflections
4449 independent reflections
3475 reflections with *I* > 2σ(*I*)
*R*_int_ = 0.050

#### Refinement

*R*[*F* ^2^ > 2σ(*F* ^2^)] = 0.042
*wR*(*F* ^2^) = 0.080
*S* = 1.09
4449 reflections
190 parameters
H atoms treated by a mixture of independent and constrained refinement
Δρ_max_ = 1.92 e Å^−3^
Δρ_min_ = −0.86 e Å^−3^

### 

Data collection: *APEX2* (Bruker, 2005 ▶); cell refinement: *SAINT* (Bruker, 2005 ▶); data reduction: *SAINT*; program(s) used to solve structure: *SHELXTL* (Sheldrick, 2008 ▶); program(s) used to refine structure: *SHELXTL*; molecular graphics: *SHELXTL*; software used to prepare material for publication: *SHELXTL* and *PLATON* (Spek, 2009 ▶).

## Supplementary Material

Crystal structure: contains datablock(s) global, I. DOI: 10.1107/S1600536811052433/is5025sup1.cif

Structure factors: contains datablock(s) I. DOI: 10.1107/S1600536811052433/is5025Isup2.hkl

Supplementary material file. DOI: 10.1107/S1600536811052433/is5025Isup3.cml

Additional supplementary materials:  crystallographic information; 3D view; checkCIF report

## Figures and Tables

**Table 1:** Hydrogen-bond geometry (Å, °)

*D*—H⋯*A*	*D*—H	H⋯*A*	*D*⋯*A*	*D*—H⋯*A*
C14—H14*A*⋯I1^i^	0.93	3.00	3.915 (3)	169
C17—H17*A*⋯I1^ii^	0.93	2.93	3.840 (3)	167

Symmetry codes: (i) 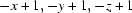; (ii) 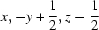.

**Fig. 1.**
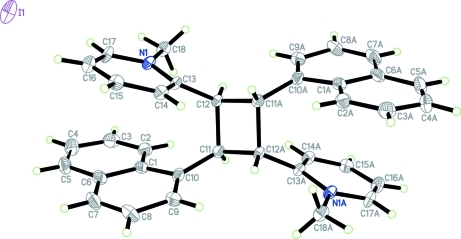
The molecular structure of the title compound, with 50% probability displacement ellipsoids and the atom-numbering scheme. The suffix A corresponds to symmetry code 1 - x, 1 - y, 1 - z.

**Fig. 2.**
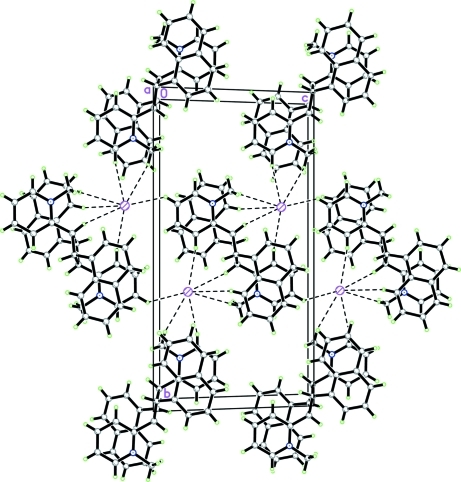
The crystal packing of the title compound viewed down the a axis. C—H···I interactions are shown as dashed lines.

## supplementary crystallographic information

### Comment

Stilbene derivatives have been reported to exhibit non-linear optical (NLO) property (Ruanwas *et al.*, 2010) and antibacterial activity (Chanawanno *et al.*, 2010). It has led us to investigate the bioactivity of [2 + 2] cycloaddition product of stibene derivatives. In general the [2 + 2] dimerization of stilbene can occurred by photoinduced cycloaddition reaction (Papaefstathiou *et al.*, 2002). In our case, however, the [2,2'-(2,4-di(naphthalen-1-yl)cyclobutane-1,3-diyl)bis(1-methylpyridinium)] diiodide, compound (I), was produced by thermal-induced [2 + 2] cycloaddition reaction of (*E*)-1-methyl-2-[2-(1-naphthyl)vinyl)pyridinium iodide in hot methanol at 323 K. We have also previously reported the crystal structures of the [2 + 2] cycloaddition compounds (Fun, Chanawanno and Chantrapromma, 2009; Fun, Surasit *et al.*, 2009).

The molecular structure of the title compound consists of one C_36_H_32_N_2_^2+^ cation and two I^-^ anions (Fig. 1). The cation lie on and the anion lie near an inversion center. The naphthalene (C1–C10) ring system is planar with an *r.m.s*. deviation of 0.0479 (4) Å. The dihedral angle between the pyridine (N1/C13–C17) ring and the naphthalenyl ring system is 19.01 (14)°. The steroisomer of (I) is *syn* head-to-tail (Yayli *et al.*, 2004), and the torsion angle C10–C11–C12–C13 = 1.8 (4)°. The cyclobutane ring makes the dihedral angles of 88.1 (2), 75.9 (2) and 70.8 (2)° with the N1/C13–C17, C1–C6 and C1/C6–C10 rings, respectively. The bond lengths in cation are in normal ranges (Allen *et al.*, 1987) and comparable with those in related structures (Fun, Surasit *et al.*, 2009; Fun, Chanawanno & Chantrapromma, 2009).

The crystal packing of (I) is shown in Fig. 2. The anions are located in the interstitials of the cations and linked with the cations into three-dimensional network by C—H···I interactions (Table 1). π–π interactions were presented with distances of *Cg*1···*Cg*2 = 3.580 (2) Å, *Cg*1···*Cg*3 = 3.533 (2) Å, *Cg*1···*Cg*2^iii, iv^ = 3.807 (2) Å [symmetry codes: (iii) -1 + *x*, *y*, *z*; (iv) 1 + *x*, *y*, *z*]; *Cg*1, *Cg*2 and *Cg*3 are the centroids of N1/C13–C17, C1–C6 and C1/C6–C10 rings, respectively.

### Experimental

A solution of (*E*)-1-methyl-2-[2-(1-naphthyl)vinyl)pyridinium iodide (500 mg) in CH_3_OH (20 ml) was heated at 323 K until a clear solution was obtained and then left to stand at room temperature overnight. The yellow powder which is the product of [2 + 2] cycloaddition reaction of heterostilbene was formed. Yellow block-shaped single crystals of compound (I) suitable for *X*-ray structure determination were obtained after recrystallization in CH_3_OH by slow evaporation of the solvent at room temperature after a few weeks.

### Refinement

H atoms of cyclobutane (at atom C11 and C12) are located in difference maps and refined isotropically. The remaining H atoms were positioned geometrically and allowed to ride on their parent atoms, with *d*(C—H) = 0.93 Å for aromatic and 0.96 Å for CH_3_ atoms. The *U*_iso_(H) values were constrained to be 1.5*U*_eq_ of the carrier atom for methyl H atoms and 1.2*U*_eq_ for the remaining H atoms. A rotating group model was used for the methyl groups. The highest residual electron density peak is located at 0.89 Å from I1 and the deepest hole is located at 1.21 Å from H18C.

### Crystal data

**Table:**

C_36_H_32_N_2_^2+^·2I^−^	*F*(000) = 736
*M**_r_* = 746.44	*D*_x_ = 1.625 Mg m^−^^3^
Monoclinic, *P*2_1_/*c*	Mo *K*α radiation, λ = 0.71073 Å
Hall symbol: -P 2ybc	Cell parameters from 4449 reflections
*a* = 7.0061 (1) Å	θ = 2.2–30.0°
*b* = 20.7920 (4) Å	µ = 2.09 mm^−^^1^
*c* = 10.8956 (2) Å	*T* = 100 K
β = 106.063 (1)°	Block, yellow
*V* = 1525.21 (5) Å^3^	0.15 × 0.13 × 0.08 mm
*Z* = 2	

### Data collection

**Table:**

Bruker APEXII CCD area-detector diffractometer	4449 independent reflections
Radiation source: sealed tube	3475 reflections with *I* > 2σ(*I*)
graphite	*R*_int_ = 0.050
φ and ω scans	θ_max_ = 30.0°, θ_min_ = 2.2°
Absorption correction: multi-scan (*SADABS*; Bruker, 2005)	*h* = −9→9
*T*_min_ = 0.749, *T*_max_ = 0.854	*k* = −22→29
18762 measured reflections	*l* = −15→15

### Refinement

**Table:**

Refinement on *F*^2^	Primary atom site location: structure-invariant direct methods
Least-squares matrix: full	Secondary atom site location: difference Fourier map
*R*[*F*^2^ > 2σ(*F*^2^)] = 0.042	Hydrogen site location: inferred from neighbouring sites
*wR*(*F*^2^) = 0.080	H atoms treated by a mixture of independent and constrained refinement
*S* = 1.09	*w* = 1/[σ^2^(*F*_o_^2^) + (0.0278*P*)^2^ + 1.442*P*] where *P* = (*F*_o_^2^ + 2*F*_c_^2^)/3
4449 reflections	(Δ/σ)_max_ = 0.001
190 parameters	Δρ_max_ = 1.92 e Å^−^^3^
0 restraints	Δρ_min_ = −0.86 e Å^−^^3^

### Special details

**Table:**

Experimental. The crystal was placed in the cold stream of an Oxford Cryosystems Cobra open-flow nitrogen cryostat (Cosier & Glazer, 1986) operating at 100.0 (1) K.
Geometry. All e.s.d.'s (except the e.s.d. in the dihedral angle between two l.s. planes) are estimated using the full covariance matrix. The cell e.s.d.'s are taken into account individually in the estimation of e.s.d.'s in distances, angles and torsion angles; correlations between e.s.d.'s in cell parameters are only used when they are defined by crystal symmetry. An approximate (isotropic) treatment of cell e.s.d.'s is used for estimating e.s.d.'s involving l.s. planes.
Refinement. Refinement of *F*^2^ against ALL reflections. The weighted *R*-factor *wR* and goodness of fit *S* are based on *F*^2^, conventional *R*-factors *R* are based on *F*, with *F* set to zero for negative *F*^2^. The threshold expression of *F*^2^ > σ(*F*^2^) is used only for calculating *R*-factors(gt) *etc*. and is not relevant to the choice of reflections for refinement. *R*-factors based on *F*^2^ are statistically about twice as large as those based on *F*, and *R*- factors based on ALL data will be even larger.

### Fractional atomic coordinates and isotropic or equivalent isotropic displacement parameters (Å^2^)

**Table:**

	*x*	*y*	*z*	*U*_iso_*/*U*_eq_	
I1	0.51619 (4)	0.361747 (10)	0.802649 (19)	0.02498 (7)	
N1	0.5483 (4)	0.35243 (12)	0.3555 (2)	0.0160 (5)	
C1	0.0993 (5)	0.40695 (16)	0.3501 (3)	0.0195 (6)	
C2	0.1492 (5)	0.35697 (16)	0.4416 (3)	0.0235 (7)	
H2A	0.2212	0.3664	0.5250	0.028*	
C3	0.0915 (5)	0.29415 (17)	0.4080 (4)	0.0291 (8)	
H3A	0.1233	0.2618	0.4692	0.035*	
C4	−0.0154 (5)	0.27911 (19)	0.2811 (4)	0.0347 (9)	
H4A	−0.0477	0.2366	0.2576	0.042*	
C5	−0.0712 (5)	0.3272 (2)	0.1930 (4)	0.0313 (9)	
H5A	−0.1445	0.3170	0.1103	0.038*	
C6	−0.0201 (5)	0.39223 (18)	0.2246 (3)	0.0241 (8)	
C7	−0.0847 (5)	0.44256 (19)	0.1352 (3)	0.0276 (8)	
H7A	−0.1624	0.4331	0.0532	0.033*	
C8	−0.0340 (5)	0.50453 (19)	0.1684 (3)	0.0281 (8)	
H8A	−0.0860	0.5375	0.1113	0.034*	
C9	0.0972 (5)	0.51915 (17)	0.2890 (3)	0.0226 (7)	
H9A	0.1347	0.5617	0.3086	0.027*	
C10	0.1710 (5)	0.47208 (15)	0.3781 (3)	0.0182 (6)	
C11	0.3468 (5)	0.48207 (15)	0.4937 (3)	0.0168 (6)	
C12	0.5522 (5)	0.45067 (14)	0.4853 (3)	0.0145 (6)	
C13	0.5385 (4)	0.41782 (15)	0.3610 (3)	0.0153 (6)	
C14	0.4890 (5)	0.45169 (15)	0.2464 (3)	0.0174 (6)	
H14A	0.4889	0.4964	0.2474	0.021*	
C15	0.4397 (5)	0.41930 (16)	0.1308 (3)	0.0203 (7)	
H15A	0.4098	0.4422	0.0545	0.024*	
C16	0.4353 (5)	0.35306 (16)	0.1291 (3)	0.0229 (7)	
H16A	0.3934	0.3308	0.0523	0.027*	
C17	0.4939 (5)	0.32046 (16)	0.2430 (3)	0.0215 (7)	
H17A	0.4961	0.2757	0.2427	0.026*	
C18	0.6237 (5)	0.31341 (15)	0.4735 (3)	0.0203 (7)	
H18A	0.7586	0.3253	0.5149	0.030*	
H18B	0.6182	0.2686	0.4515	0.030*	
H18C	0.5430	0.3211	0.5303	0.030*	
H11	0.305 (5)	0.4670 (18)	0.574 (3)	0.026 (10)*	
H12	0.610 (5)	0.4215 (17)	0.560 (3)	0.019 (9)*	

### Atomic displacement parameters (Å^2^)

**Table:**

	*U*^11^	*U*^22^	*U*^33^	*U*^12^	*U*^13^	*U*^23^
I1	0.04756 (15)	0.01318 (10)	0.01593 (10)	−0.00056 (11)	0.01167 (8)	0.00136 (9)
N1	0.0222 (13)	0.0125 (13)	0.0156 (12)	0.0010 (11)	0.0089 (10)	0.0007 (10)
C1	0.0145 (15)	0.0240 (17)	0.0214 (16)	−0.0010 (13)	0.0071 (12)	−0.0014 (13)
C2	0.0201 (16)	0.0197 (16)	0.0315 (18)	−0.0017 (14)	0.0082 (13)	−0.0016 (14)
C3	0.0250 (19)	0.0199 (17)	0.044 (2)	−0.0014 (15)	0.0115 (16)	0.0018 (15)
C4	0.027 (2)	0.0250 (19)	0.051 (3)	−0.0103 (16)	0.0085 (18)	−0.0148 (17)
C5	0.0227 (18)	0.036 (2)	0.034 (2)	−0.0104 (16)	0.0056 (16)	−0.0149 (17)
C6	0.0143 (15)	0.0321 (19)	0.0263 (19)	−0.0038 (14)	0.0065 (14)	−0.0053 (14)
C7	0.0197 (17)	0.042 (2)	0.0186 (17)	−0.0033 (16)	0.0007 (13)	−0.0016 (15)
C8	0.0182 (17)	0.036 (2)	0.0262 (18)	−0.0011 (15)	−0.0005 (14)	0.0061 (15)
C9	0.0202 (16)	0.0238 (17)	0.0238 (17)	0.0014 (14)	0.0059 (13)	0.0006 (13)
C10	0.0198 (16)	0.0185 (15)	0.0176 (15)	−0.0016 (13)	0.0075 (12)	−0.0048 (12)
C11	0.0186 (15)	0.0155 (14)	0.0165 (14)	0.0007 (12)	0.0053 (12)	−0.0011 (11)
C12	0.0182 (15)	0.0132 (14)	0.0131 (14)	0.0001 (12)	0.0061 (11)	−0.0011 (11)
C13	0.0190 (15)	0.0127 (14)	0.0151 (14)	0.0009 (12)	0.0061 (11)	−0.0017 (12)
C14	0.0206 (16)	0.0144 (15)	0.0184 (14)	−0.0002 (12)	0.0073 (12)	0.0019 (12)
C15	0.0220 (16)	0.0226 (16)	0.0170 (15)	0.0006 (14)	0.0068 (12)	0.0028 (13)
C16	0.0292 (18)	0.0215 (18)	0.0185 (15)	−0.0012 (14)	0.0075 (13)	−0.0028 (13)
C17	0.0326 (19)	0.0131 (15)	0.0206 (15)	−0.0014 (14)	0.0104 (14)	−0.0053 (12)
C18	0.0285 (18)	0.0146 (15)	0.0179 (15)	0.0034 (13)	0.0069 (13)	0.0037 (12)

### Geometric parameters (Å, °)

**Table:**

N1—C17	1.354 (4)	C9—H9A	0.9300
N1—C13	1.363 (4)	C10—C11	1.513 (4)
N1—C18	1.488 (4)	C11—C12^i^	1.555 (4)
C1—C2	1.415 (5)	C11—C12	1.606 (4)
C1—C6	1.424 (4)	C11—H11	1.05 (4)
C1—C10	1.447 (4)	C12—C13	1.496 (4)
C2—C3	1.386 (5)	C12—C11^i^	1.555 (4)
C2—H2A	0.9300	C12—H12	1.01 (3)
C3—C4	1.413 (5)	C13—C14	1.392 (4)
C3—H3A	0.9300	C14—C15	1.385 (4)
C4—C5	1.365 (6)	C14—H14A	0.9300
C4—H4A	0.9300	C15—C16	1.377 (5)
C5—C6	1.417 (5)	C15—H15A	0.9300
C5—H5A	0.9300	C16—C17	1.373 (5)
C6—C7	1.416 (5)	C16—H16A	0.9300
C7—C8	1.359 (5)	C17—H17A	0.9300
C7—H7A	0.9300	C18—H18A	0.9600
C8—C9	1.413 (5)	C18—H18B	0.9600
C8—H8A	0.9300	C18—H18C	0.9600
C9—C10	1.375 (4)		
			
C17—N1—C13	121.6 (3)	C10—C11—C12^i^	118.6 (3)
C17—N1—C18	117.3 (3)	C10—C11—C12	115.6 (2)
C13—N1—C18	121.1 (3)	C12^i^—C11—C12	89.7 (2)
C2—C1—C6	118.9 (3)	C10—C11—H11	108 (2)
C2—C1—C10	122.3 (3)	C12^i^—C11—H11	112 (2)
C6—C1—C10	118.7 (3)	C12—C11—H11	113 (2)
C3—C2—C1	120.6 (3)	C13—C12—C11^i^	117.1 (3)
C3—C2—H2A	119.7	C13—C12—C11	113.8 (2)
C1—C2—H2A	119.7	C11^i^—C12—C11	90.3 (2)
C2—C3—C4	120.2 (4)	C13—C12—H12	111.6 (19)
C2—C3—H3A	119.9	C11^i^—C12—H12	111 (2)
C4—C3—H3A	119.9	C11—C12—H12	111.4 (19)
C5—C4—C3	119.8 (3)	N1—C13—C14	117.9 (3)
C5—C4—H4A	120.1	N1—C13—C12	120.3 (3)
C3—C4—H4A	120.1	C14—C13—C12	121.3 (3)
C4—C5—C6	121.6 (3)	C15—C14—C13	120.5 (3)
C4—C5—H5A	119.2	C15—C14—H14A	119.8
C6—C5—H5A	119.2	C13—C14—H14A	119.8
C7—C6—C5	121.8 (3)	C16—C15—C14	119.8 (3)
C7—C6—C1	119.5 (3)	C16—C15—H15A	120.1
C5—C6—C1	118.7 (3)	C14—C15—H15A	120.1
C8—C7—C6	120.5 (3)	C17—C16—C15	118.8 (3)
C8—C7—H7A	119.8	C17—C16—H16A	120.6
C6—C7—H7A	119.8	C15—C16—H16A	120.6
C7—C8—C9	120.4 (3)	N1—C17—C16	121.0 (3)
C7—C8—H8A	119.8	N1—C17—H17A	119.5
C9—C8—H8A	119.8	C16—C17—H17A	119.5
C10—C9—C8	121.6 (3)	N1—C18—H18A	109.5
C10—C9—H9A	119.2	N1—C18—H18B	109.5
C8—C9—H9A	119.2	H18A—C18—H18B	109.5
C9—C10—C1	118.4 (3)	N1—C18—H18C	109.5
C9—C10—C11	123.4 (3)	H18A—C18—H18C	109.5
C1—C10—C11	117.3 (3)	H18B—C18—H18C	109.5
			
C6—C1—C2—C3	3.3 (5)	C1—C10—C11—C12^i^	170.7 (3)
C10—C1—C2—C3	−174.5 (3)	C9—C10—C11—C12	−103.7 (4)
C1—C2—C3—C4	1.0 (5)	C1—C10—C11—C12	65.9 (4)
C2—C3—C4—C5	−3.5 (6)	C10—C11—C12—C13	1.8 (4)
C3—C4—C5—C6	1.6 (6)	C12^i^—C11—C12—C13	−120.0 (3)
C4—C5—C6—C7	−177.3 (3)	C10—C11—C12—C11^i^	121.9 (3)
C4—C5—C6—C1	2.6 (5)	C12^i^—C11—C12—C11^i^	0.0
C2—C1—C6—C7	174.9 (3)	C17—N1—C13—C14	−6.2 (4)
C10—C1—C6—C7	−7.3 (5)	C18—N1—C13—C14	172.5 (3)
C2—C1—C6—C5	−5.0 (5)	C17—N1—C13—C12	165.3 (3)
C10—C1—C6—C5	172.8 (3)	C18—N1—C13—C12	−16.0 (4)
C5—C6—C7—C8	179.8 (3)	C11^i^—C12—C13—N1	147.3 (3)
C1—C6—C7—C8	−0.1 (5)	C11—C12—C13—N1	−109.3 (3)
C6—C7—C8—C9	5.2 (5)	C11^i^—C12—C13—C14	−41.5 (4)
C7—C8—C9—C10	−2.7 (5)	C11—C12—C13—C14	61.9 (4)
C8—C9—C10—C1	−4.8 (5)	N1—C13—C14—C15	3.8 (5)
C8—C9—C10—C11	164.7 (3)	C12—C13—C14—C15	−167.6 (3)
C2—C1—C10—C9	−172.6 (3)	C13—C14—C15—C16	1.5 (5)
C6—C1—C10—C9	9.6 (5)	C14—C15—C16—C17	−4.6 (5)
C2—C1—C10—C11	17.3 (4)	C13—N1—C17—C16	3.1 (5)
C6—C1—C10—C11	−160.5 (3)	C18—N1—C17—C16	−175.6 (3)
C9—C10—C11—C12^i^	1.1 (5)	C15—C16—C17—N1	2.4 (5)

Symmetry codes: (i) −*x*+1, −*y*+1, −*z*+1.

### Hydrogen-bond geometry (Å, °)

**Table:**

*D*—H···*A*	*D*—H	H···*A*	*D*···*A*	*D*—H···*A*
C14—H14A···I1^i^	0.93	3.00	3.915 (3)	169
C17—H17A···I1^ii^	0.93	2.93	3.840 (3)	167

Symmetry codes: (i) −*x*+1, −*y*+1, −*z*+1; (ii) *x*, −*y*+1/2, *z*−1/2.
